# Abstract of the Proceedings of the Buffalo Medical Association

**Published:** 1863-11

**Authors:** J. F. Miner

**Affiliations:** Secretary


ART. II.—*Abstract of the Proceedings of the Buffalo Medical Association.*

TUESDAY EVENING, Oct. 6, 1863.

Dr. Congar, President, in the Chair.

Minutes of the last meeting read and corrected—*Dr. Rochester* saying that he spoke of the Acid Treatment in diarrhœa and not in dysentery and *Dr. Wyckoff* remarked that the case seen by him, with Dr. Storck, was one with all the symptoms of cholera, rice colored stools, cramps, cold surface, collapse, etc., these symptoms rapidly succeeding to the dysenteric discharges; this peculiarity alone constituted the interest of the case. Approved as corrected.

*Dr. Miner* introduced a patient with Melanosis of the eye-ball, and gave the following history: Mr. L., aged 49 years; observed imperfect vision in right eye about one year since, which gradually increased until in a few months he had distinct cataract in that eye; and soon after the opacity of the lens became visible, there also appeared a dark spot on the inner side of the globe a few lines posterior to the junction of the cornea with the sclerotica. This at first attracted very little attention, and appeared very much like a small varix of vessels in the conjunctival membrane. July 7th, operation was made for division and depression of the lens, which produced no inflammatory disturbance of the eye, and for a time promised favorable result. August 3d, the dark spot in the conjunctiva had grown perceptibly, and effort was made to remove it. It was found to extend into sclerotic coat, and to be beyond the reach of removal. The lens and capsule have since become involved, and now it will be observed that the iris is changed, thickened and blackened. The membranes of the globe and the contents of the eye are all involved in melanotic disease.

This patient had been examined by quite a number of distinguished surgeons while the disease was in its incipient stage; and the patient informed that it was a very rare case, and that they were unable to determine its character. Some of the oldest and most experienced surgeons in the State told him that they had never seen similar disease, and did not know what it was. It appears a black degeneration of the tissues of the eye, rapidly extending its borders, producing no pain or inconvenience other than loss of sight; and opacity of the lens had previously produced total blindness, so that the melanosis cannot fairly be said to have caused loss of vision. If it is not melanotic disease, the question still remains; what is it? At first it might have been regarded as staphyloma or hernia of the choroid, or pigmentary tunic of the eye; but its rapid extension to the lens and discoloration of the contents of the globe prove that it is not of such nature, and leave no doubt as to the true character of the disease.

It has been presented before the Society because it is a rare form of malignant degeneration, and supposed to be of interest to those who have never observed it, or have never seen it in its earlier and formative stage. When it shall become a protruding blackened mass, it will lose its interest to the surgeon, and remain only an incurable and fatal disease. The only rational advice which can be given in the case as it now presents itself is, extirpation of the globe of the eye. This advice will be given this patient as soon as the disease is so fully established as to leave no grounds for doubt, and in his opinion that period had already arrived. It cannot, however, be urged with perfect assurance, that even this will overcome the disease. There is not (as is most common) melanotic disease in other parts of the body, so far as is known; if there is, it is of internal organs, and gives no symptoms indicating it. Its removal may be attended by favorable results; there are sufficient grounds of expectation in this case, at least, to justify the measure. It is thought to be less liable to return and less rapid in its march than encephaloid, yet it is something of the same character, and leads to the same results.

*Dr. White* said he had seen two cases of melanosis, in one of which he removed the eye: the patient died the following year of fungus hæmatodes.

*Dr. Shaw* mentioned a case which he had seen, where there was large tumor and no appearance of the natural eye.

*Dr. Ring* recollected a case presented at the clinic while he attended lectures in Geneva. The eye was extirpated, and he thinks the disease did not return.

*Dr. Rochester* remarked that since Dr. Miner had introduced the subject of diseases of the eye, he would relate some of the particulars of one or two interesting cases which had recently fallen to his care. A gentleman called upon him not long since, saying that he had come to tell him that he was blind in one eye; though the eye looked naturally, he could not see. He had received a blow upon the eye. Upon examination, after the pupil was dilated, could distinctly see opake lens. The blow had produced traumatic cataract.

Another man, while chopping wood, had a stick fly up and strike him in the eye; he fainted from the effects of the blow. When seen the pupil was dilated, and there was no contraction from effects of light. The next day it had not improved, and there was more occasion for fearing amauro sis. He was treated with blisters, cathartics, cold applications and Iodide of Potassium. This case finally recovered.

A third similar case was that of a woman, who had a beer-bottle cork fly against her eye, which also appeared to have produced traumatic cataract and also amaurosis. The amaurosis, or loss of function in the nerve, is supposed to be produced by the direct injury of the blow upon the nervous structure, while the inflammatory action or stasis which this induces, is probably the cause of the opacity in the lens.

*Dr. Shaw* related a case struck with a ball-club, which produced dilatation of the pupil, which still remains, yet vision is improving.

*Dr. Cronyn* related similar cases; one was struck by a stick of wood; the pupil was dilated. Cold water was applied, and the pupil gradually contracted; but there was no return of vision. Another similar case, attended with high inflammatory action, was treated actively, with the effect of subduing the inflammation, but not of restoring vision.

*Dr. White*, while upon the subject of eyes, would call attention to the practice when foreign substances are lodged in the posterior chamber of the eye. Bits of steel or portions of percussion cap are the more common substances. It is plainly in sight, and the impulse both with physician and friends, is to remove it. In such cases, should anything be done when it cannot be seized and removed through the opening which itself has made? A few days since a lady from Chautauqua county, brought a little girl to him, with part of a percussion cap in the posterior chamber of the eye. It could be distinctly seen, and she had been advised by her physician to visit the city to have it removed Thought more irritation would be produced in this instance by the removal than it would be likely to produce if let alone, and advised rest, exclusion of light, cold applications, and an aperient, and had since learned that inflammation had abated under that treatment. Vision was imperfect, inflammation and pain moderately severe; and he thought that if operation had been made, possibly the eyeball would have been spoiled. Was satisfied in his own mind that if it could not be taken out from the opening it had itself made, it should not be removed. This case was seen also by Drs. Rochester, Eastman and Boardman, who coincided in the opinion he had expressed. The responsibility in such cases was sometimes great, and physicians might say, how stupid not to remove it! It required some courage to advise not to interfere. This case had been related and these remarks made for the purpose of placing on record, that such substances may often better be left than to run the risks attending attempts at removal.

*Dr. Cronyn* said it was remarkable what prejudice would do in some such instances, There had recently been a discussion in London upon the very subject. It used to be thought necessary to remove in order to save the opposite eye, but it is clearly stated in the *Lancet* report of the discussion that such substances should not be removed unless they are producing inflammation.

Has now a case where a piece of steel is unquestionably in the globe of the eye. It produces no great distubance; the globe is a little atrophied.

Also related a case where a shot had been lodged in the posterior chamber of the eye. He was called, but before his arrival they had made ready for the operation. An old man, who had been a medical student and schoolmaster in Glasgow, had informed them, that if the Doctor knew anything he could turn the eye out and remove the shot immediately. The man was in amazement when he introduced a currette and displaced it into the vitrious humor, especially when pain, which had been severe, immediately subsided. This substance could be distinctly felt with curette, yet no effort was made to extract it. So strong was the impression made upon the minds of the friends by the school-master, that several physicians were consulted before they became satisfied as to the correctness of the practice.

Drs. White, Ring, Cronyn, Wyckoff, Rochester and others, spoke of the prevalence of pulmonary disease and scarlet fever, and also some cases of diphtheria.

*Dr. Miner* had nothing to say of prevailing diseases, but desired to make a remark or two upon the subject introduced by Dr. White, though this had been passed in the order of exercises. Dr. White and others had recited cases where foreign substances had been left in the eye and so far as known no very serious consequences had followed. The final results of these cases are as yet undetermined, and if known to be favorable show, only that in these instances such practice was justifiable and safe. Without having had his attention called to this subject with the view to learn what may have been the general opinions of authors of Ophthalmic Surgery, he was yet confident that surgeons had as a rule regarded the removal of such substances, when their positions could be distinctly discoverable as much more safe and proper than trusting to the expectation of their remaining harmless. Though Dr. Cronyn had informed us, that in the *Lancet*, this principle had recently been settled, yet he could not think that any good authority could previous to this, be found to sustain the position that foreign bodies in plain sight in the eye should not be removed unless they can be taken from the opening made by their entrance within the globe.

This suggestion from Dr. White was a very important one, and though he did not desire to express a doubt but that the case from the country received most judicious advice, and that other cases related were properly treated, still he thought in many similar instances a skillful operator, with proper instruments and at favorable time, might remove such foreign substance and obtain better results than if left to the uncertainties of spontaneous cure.

*Dr. Rochester* desired to call the attention of the editor of the *Medical Journal* to the advertisement of Wade & Ford, of New York, with the attachment "Sole Agents for Firmenich's Irritation Instrument," thus giving those who are unacquainted the idea that it is something. That such thing should by any process obtain mention in the advertising sheet of the *Buffalo Medical Journal*, was quite surprising to him, and he presumed the editor never had his attention called to it, for it is not long since, he condemned the practice in other medical journals of advertising to the profession an instrument possessing no advantages, whose use was claimed and quackishly applied, by an irregular practioner, as a sovereign remedy for all diseases.

*Dr. Miner* replied that the publisher of the *Journal* did not vouch for the goods advertised. If that was the idea of any one, they were mistaken. Every careful reader of the *Journal* knew that the Editor regarded Firmenich's Instrument as good for nothing. He was sorry that a firm manufacturing the best surgical instruments in this country should append to their card "Agents" for any such thing, and was sure that if they were well acquainted in Buffalo and vicinity it would be the last thing they would be agents for; and, if agents, would desire to have it known as little as possible. Was glad the subject had been introduced, thus giving opportunity for explanation.

*Voted* to adjourn. J. F. MINER, *Secretary*.

