# Correction in Last Month’s Report of the Discussion upon Injuries of the Eye

**Published:** 1863-12

**Authors:** 


CORRECTION IN LAST MONTH'S REPORT OF THE DISCUSSION UPON INJURIES OF THE EYE.—Dr. Cronyn related similar cases, *one* of which corresponded with that related by Dr. Rochester, a stick of wood striking globe of eye, producing faintness, sickness at stomach, &c. Came to office some days after injury with permanently dilated pupil and loss of vision, which still continued. Another case, that of a young lad 17 years old, injured in stave factory, was seen immediately and treated actively by leeching, cold, &c., and pupil gradually contracted and vision is now quite restored.

Again, this subject was recently discussed in *Lancet* and decided that when a foreign body produces no irritation and is beyond reach in *posterior* chamber of eye, it was better to let it alone, but if it produced irritation at any time and the foreign body could not be removed, lest the sound eye should suffer, the *injured* one ought to be removed."

